# Electricity in the Diagnosis and Treatment of Diseases of the Nose, Throat and Ear

**Published:** 1898-12

**Authors:** 


Electricity in the Diagnosis and Treatment of Diseases of the Nose, Throat and Ear. By W. Scheppegrell, A. M., M. D., Ex-vice-president American Laryngological, Rhinological and Otological Society; Vice-president Western Ophthalmologic and Oto-laryngologic Association; Late Assistant Surgeon to the Eye, Ear, Nose and Throat Hospital, New Orleans; Vice-president New Orleans Electric Society; Co-editor Annals of Otology, Rhinology and Laryngology; Associate Editor The Laryngoscope; Collaborator The Revue Internationale de Rhinologie, Otologie, et Laryngologie; Member American Academy of Medicine, etc., New Orleans, La. Octavo, 403 pages, 161 illustrations. New York: G. P. Putnam's Sons. 1898.

The author presents in this book a very complete survey of the uses and application of electricity in the diagnosis and treatment of diseases of the ear, nose and throat, including the means of generating and measuring it and a description of the instruments used. The literature of the subject has been liberally drawn upon, to which the author has added many original suggestions. Among the subjects carefully considered, the following are of special practical interest—namely, illumination, trans-illumination, the electro-cautery, electrolysis, cataphoresis and auditory electro-massage. The work is a most useful contribution to this department of electro-therapeutics.

